# Sprint to work: A novel model for team science collaboration in academic medicine

**DOI:** 10.1007/s40037-018-0442-9

**Published:** 2018-07-23

**Authors:** Shashank S. Sinha, Tedi A. Engler, Brahmajee K. Nallamothu, Andrew M. Ibrahim, Ann Verhey-Henke, Marianna Kerppola, Chandy Ellimoottil, Andrew M. Ryan

**Affiliations:** 1 Medical School, University of Michigan, Ann Arbor, MI USA; 2 Center for Evaluating Health Reform, School of Public Health, University of Michigan, Ann Arbor, MI USA; 3 Division of Cardiovascular Diseases, Department of Internal Medicine, Medical School, University of Michigan, Ann Arbor, MI USA; 4 School of Public Health, University of Michigan, Ann Arbor, MI USA; 5 Department of Urology, Medical School, University of Michigan, Ann Arbor, MI USA

**Keywords:** Team processes, Academic medicine, Health services research

## Abstract

Collaborative research in academic medicine is often inefficient and ineffective. It often fails to leverage the expertise of interdisciplinary team members, does not seek or incorporate team input at opportune times, and creates workload inequities. Adapting approaches developed in venture capital, we created the ‘sprint model’ for writing academic papers based on the analysis of secondary data. The ‘sprint model’ minimizes common barriers that undermine collaboration in academic medicine. This model for team science collaboration begins with team members convening for a highly focused, guided session. In this session, a facilitator moves the group through a structured process to create the study plan. This includes refining the research questions, developing the study design, and prototyping the presentation of results. After adopting this model, our team has drastically reduced time from idea inception to final product submission through increased efficiencies and reduced redundancies. From December 2016 to April 2018, our team has initiated 15 paper sprints. The median time from sprint to submission for paper sprints has been 1.7 months (minimum: 0.5; maximum: 9). Although our current ‘sprint’ approach has already demonstrated a substantial improvement in our ability to rapidly produce high-quality research, we believe the ‘pre-sprint’ preparation and ‘post-sprint’ processes can be further refined. Finally, we discuss the limitations of this model and our efforts to adapt the process to meet the evolving needs of research teams.

**Electronic supplementary material:**

The online version of this article (10.1007/s40037-018-0442-9) contains supplementary material, which is available to authorized users.

## Introduction

Team science has emerged as the preeminent approach to solving complex problems in academic medicine [1–4]. However, investigators face numerous challenges in leading and fostering productive collaboration among academic teams. While there are myriad causes of failure, we postulate three important mechanisms by which the collaborative research process may be undermined [5]:

1. Wasting the time of collaborators: Investigators often seek the input of collaborators at the wrong time, usually too late. After substantial time has been sunk into the project, investigators often ignore the suggestions of collaborators that would require substantial changes to the projects. Likewise, analysts are often redirected over the course of months or years before investigators determine the study plan.
2. Failing to utilize the timely expertise of team members: Because they are often engaged too late in the project cycle, collaborators’ expertise is not optimized to formulate research questions, plan the study design and analysis, and develop the manuscript. Too often, collaborators merely make cosmetic changes to nearly finished manuscripts.
3. Creating inequities in the distribution of work: The lead investigator typically performs the vast majority of the work. This can lead to alienation, procrastination, boredom, and burnout.

As a result, collaborative research is less effective and less efficient than it could be. To address these pathologies, we developed a new model of academic collaboration—the ‘paper sprint’. This model focuses on the nuts and bolts of how teams in academic medicine can be more efficient and productive. In this paper, we describe this new model for research collaboration—developed within the framework of health services research and quantitative secondary data analysis—and illustrate the potential for making collaborative research in academic medicine more efficient, effective, and rewarding.

## Approach

Our objective was to develop and refine a structured process for team research collaboration. We were inspired by the book *Sprint*, which describes the collaborative approach invented by Google Ventures to help organizations develop, prototype, and test products within five days [6]. The academic ‘paper sprint’ is modelled on the first day of the Google Venture Sprint which is devoted to planning and seeking expert input. Google Ventures’ process is designed to tackle three main challenges that we also face in academic medicine: high stakes; limited time; and difficulty sustaining momentum [6]. With an eagerness to experiment and ‘fail fast’ [7], we extracted the project planning framework and leadership principles, adapting them to academic research.

## The nuts and bolts of paper sprints

Paper sprints are partitioned into three key phases: preparation, execution, and follow-up. Paper sprint teams typically include 4–6 people representing diverse perspectives and objectives on the project: lead investigator, senior investigator, co-investigators, fellows, students, an analyst, and a project manager. To prepare, the lead or senior investigator identifies the study question, proposes the journal that will be targeted for the first submission, outlines the basic study methods, and identifies team members, specifically, the analyst. The lead investigator also works with an analyst to produce descriptive statistics to verify that key variables required for the study are available and appropriate for the study. The project manager coordinates logistics, including arranging the time, room, agenda, and compiles and circulates meeting materials.

Also prior to the sprint, relevant literature is identified and printed so as to be available for quick *ad hoc* reference at the time of the sprint. The submission guidelines for the target journal are readily available so the team can plan a manuscript within those parameters. Similarly, preparing descriptive statistics in advance affords the opportunity to respond to limitations, building solutions into analysis plans. By compiling the materials and information teams predictably need, we can easily access these resources throughout the meeting, enabling the project team to make optimal use of everyone’s time while they are in the same room.

The paper sprint occurs over two hours. It is facilitated by the lead or senior investigator (Tab. 1). The sprint starts with the lead investigator ‘setting the stage,’ describing the manuscript title, target journal, the knowledge gaps addressed by the paper, the study methods, and what will be accomplished during the sprint. Team members have the opportunity to ask clarifying questions about the study objectives and methods. The team then collaboratively drafts the study abstract in the *Journal of the American Medical Association* (*JAMA*) style, which is easily adapted to other journals. Writing the abstract focuses the team on the kinds of inferences that we wish to make. This step is surprisingly crucial, as it often identifies a weakness in research question and study design that must be modified.

**Table 1:** Schedule for paper sprint

Element	Time (mins)	Description
Setting the stage	10	Lead investigator provides background on the sprint (2 min) – Title of the paper – Target journal – Describe theory of paper, data and constraints (3 min) a. Knowledge gaps b. Study data c. Study cohort d. Study design – What we will accomplish today
		Group responds (5 min) – Affirms or revises knowledge gaps – Asks big-picture questions about study approach
Writing the abstract	40	Use structured abstract template from JAMA
		Split into 2 teams – Intro team – Methods team
		First round of writing (15 min) – Intro team writes: Background – Methods team writes: Methods (Design, setting, participants; exposure; outcomes)
		First round of revision (5 min) – Intro team revises methods team’s work and vice versa
		Second round of writing (10 min) – Intro team writes Conclusions – Methods team writes Results
		Second round of revision (5 min) – Intro team revises methods team’s work and vice versa
Developing the exhibits	40	All team members sketch out his/her plan for the exhibits (15 min) – Use the manuscript guide for inspiration
		Potential guide – Table 1—Descriptive Stats – Table 2/Fig—Main Effects – Table 3/Fig—Heterogeneity of Effects – Table 4/Fig—Policy Impact
		Exhibit drafts are taped on the walls and reviewed (5 min)
		Team discusses the best display for exhibits (15 min)
Revising the analysis plan	20	Team reviews and comments on the analysis plan proposed (5 min)
		Team raises questions and discusses potential revisions to the analysis plan (15 min)
Determining next steps	10	Team identifies enduring weaknesses and uncertainties regarding the study plan
		Team comes up with a plan to address these issues
		Lead investigator describes the project timeline and next steps

Next, the research team works to prototype the tables and figures (‘exhibits’) that will ultimately be presented as evidence in the manuscript. To do this, each team member sketches a plan for each exhibit. The exhibits are then compiled and reviewed by the full team. The strongest exhibits are selected with modifications made based on the discussion. Using these draft exhibits as a guide, the previously outlined analysis plan is reviewed and solidified. We close by discussing enduring ambiguities, how these will be resolved, and by describing the project plan. All team members leave with clear instructions on action items, deliverables and the project timeline.

Following the initial planning sprint, the lead investigator, senior investigator, and analyst hold a meeting to further specify the analysis plan. At this point, the team has already worked through the major conceptual and intellectual issues. This enables the lead investigator and analyst to more clearly specify features of the analysis, including the inclusion criteria, study outcomes, statistical analysis, and specific statistical code.

After this meeting, the analysis is performed. Only relatively small revisions are typically required, as major methodological issues have already been resolved as a team. Once the analysis and exhibits are completed, the team typically reconvenes for a two-hour ‘writing sprint.’ During the writing sprint, the group first discusses the results and agrees on interpretation, implications, and limitations. Sections of the paper (e. g. Introduction, Methods, Results, Discussion) are then assigned to team members to draft in approximately 30 min. These sections are then rotated to another teammate for revision. This group writing process creates positive peer pressure to quickly make progress on the manuscript. Our team uses Google Docs for this process, which allows the entire team to work simultaneously on the same document. After this meeting, the lead and senior investigators work to finish the draft, which is then circulated to co-investigators for final revisions and submitted.

## Outcomes

The benefits of paper sprints have been manifold. We have reduced time to submission and the time individuals spend on a paper. From December 2016 to April 2018, our team has initiated 15 paper sprints, eight of which have been submitted to academic journals. The median time from sprint to submission has been 1.7 months (minimum: 0.5; maximum: 9). Papers written using this process have been submitted to top-tier multispecialty, generalist, and health policy journals. While the sample is small and it is still early in the experiment, this process has proven to be more efficient than our old ways of writing papers. Of the eight manuscripts submitted, three are published [8–10], three received a revise and resubmit, and two are currently under review.

## Discussion

We have been challenged in academic medicine to become more innovative, productive, and efficient, but have not had a suitable framework. We believe that our sprint process has successfully addressed many of the barriers to high-quality collaborative research in academic medicine. Our description of the sprint process is distinct from previous literature on collaborative research which has focused on broader problems in team science such as delegating tasks and setting expectations [11–17]. While important, previous work has not described a comprehensive and specific approach to improve research practice.

More importantly, the process has put the ‘team’ back in ‘team science’. By bringing project management principles to the forefront, we correct inequities in workloads. Each co-investigator contributes at a crucial time in the research process. Each team member is asked to reflect on critical issues in the design and execution of projects, maximizing individual strengths, and creating consensus around the research question and methodological approach. In this way, time, an increasingly scarce resource, is economized as decisions surrounding differences in scientific opinion are compressed to hours or minutes. By investing in intentional planning sessions, we are able to reduce frustrating and time-consuming late-stage revisions. Finally, the process has been refreshing to team members, making it possible to sustain engagement over the arduous life-cycle of a project.

The most exciting aspect of this model is its adaptability. Like any innovation, this process must adapt to the needs of users (see online Supplementary Material). Modifications of the sprint model include identifying the analyst at the outset, running descriptive statistics in advance, and engaging remote collaborators through video conferencing. We continue to fine-tune the study execution phase by implementing structured processes based on lean manufacturing principles.

We acknowledge that there are several reasons why our sprint methods may not be as effective in other circumstances. First, we have implemented these sprints within the Institute for Healthcare Policy and Innovation at the University of Michigan, which provides the co-location of researchers, access to high-quality collaborative space, data, and computational resources, and a strong culture of collaboration. However, we contend that the sprint model should be generalizable to other programs seeking to improve team processes. Second, our research has been oriented towards retrospective analysis of quantitative secondary data. Our research team has extensive experience using some of these datasets, including Medicare claims. As a result, we know in advance the possibilities and limitations of the data. It is unclear whether the sprint would be as effective in other circumstances, such as those requiring primary data collection or qualitative analysis. Finally, the sprint model benefits a skilled facilitator to elicit input from all participants [18] and guide the group to decisions.

## Conclusion

The sprint model we developed is not mystical. We believe it is consistent with many of the practices used by successful researchers. However, by codifying this model, we aim to take the alchemy out of successful collaborative work. This has allowed us to produce high-quality work more effectively and efficiently. We believe that this model can be successfully adopted by other research teams. Our successes have led to more enthusiasm about collaborative work, creating an innovative model for team science.

## Caption Electronic Supplementary Material

How to Conduct Paper Sprints. A more collaborative, efficient and fun way to write academic papers
