# Supplementary material for: Sprint to work: A novel model for team science collaboration in academic medicine
Source: Perspect Med Educ. 2018 Jul 23;7(4):281–5. doi: 10.1007/s40037-018-0442-9 (PMC6086814; doi:10.1007/s40037-018-0442-9)
Supplement: Supplementary file 1 — How to Conduct Paper Sprints. A more collaborative, efficient and fun way to write academic papers [file 40037_2018_442_MOESM1_ESM.pdf]

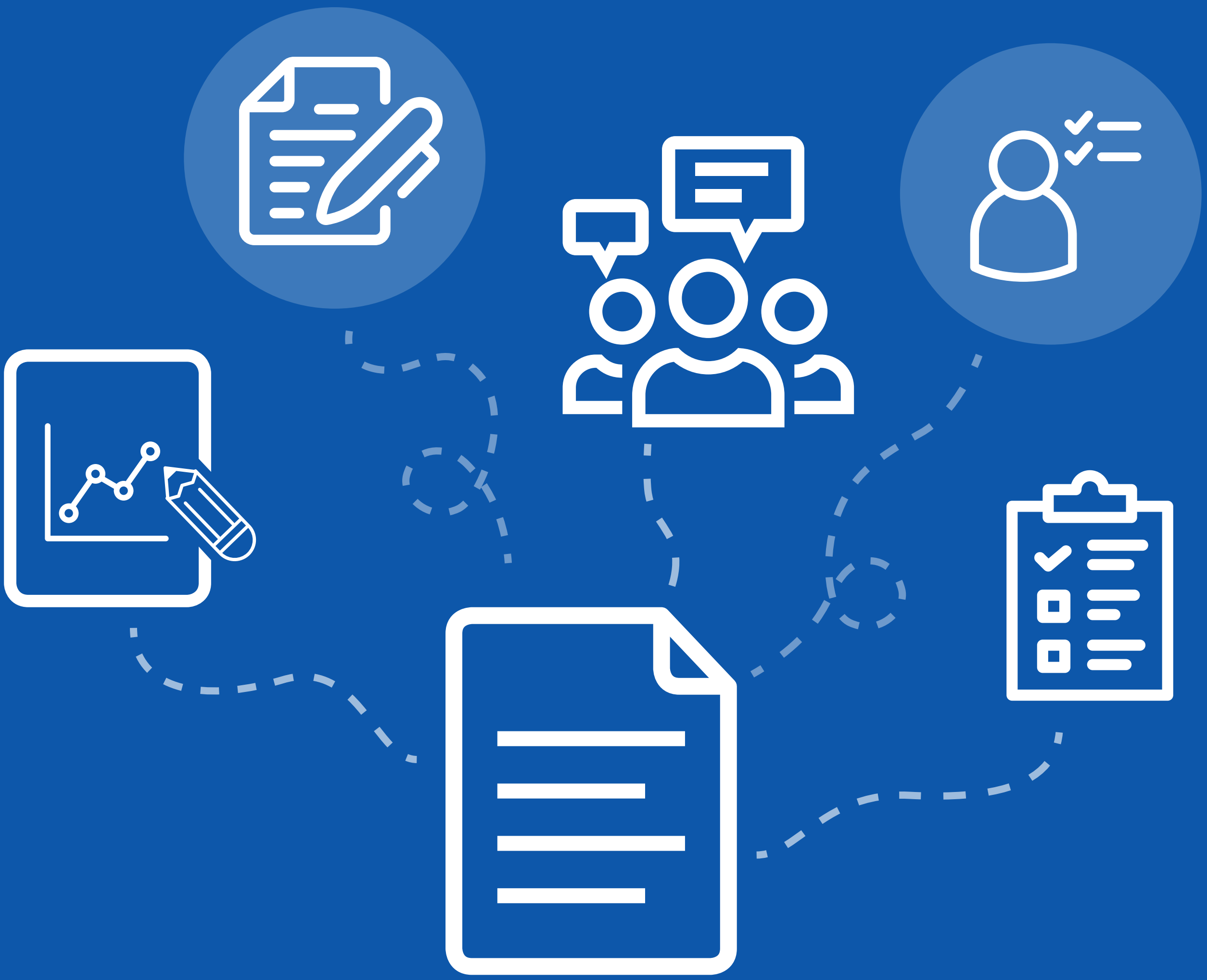

How to Conduct Paper Sprints

A more collaborative, efficient and fun way to write academic papers

SCHOOL OF PUBLIC HEALTH
CENTER FOR EVALUATING HEALTH REFORM
UNIVERSITY OF MICHIGAN

INSTITUTE FOR
HEALTHCARE POLICY & INNOVATION
UNIVERSITY OF MICHIGAN

Table of contents

Introduction	1
Getting Started	2
The Paper Sprint	7
The Writing Sprint	12

The Paper Sprint process was co-created by Andrew Ryan, Associate Professor of Health Management and Policy, Marianna Kerppola and Ann Verhey-Henke from the University of Michigan School of Public Health Innovation Studio, and colleagues from the Institute for Health Policy and Innovation.

Why Paper Sprints?

Developing academic papers – from idea conception, to analysis, to writing—can be a lonely, burdensome, and inefficient process. Deciding whether a research idea should be developed into a manuscript is hard. Spending months creating datasets and estimating models with no clear plan how these results might be used is a waste of time. Waiting for co-investigators to get back to you is annoying. Staring at a blank Word document with various half-baked ideas can feel agonizing.

We believe there is a better way to write academic papers. We have developed Paper Sprints, a novel method to write academic papers collaboratively in order to synthesize the ideas from the entire research team. By bringing together all of the collaborators in a room, the team is able to focus deeply on the research topic to maximize the quality of the research. Within a two-hour session, a group of 4–8 participants can create a fully specified project plan. If done well, after the session, all that is required is for the team to follow the plan they've outlined for themselves to execute the analysis. The group may come back together for a two-hour writing sprint, or the lead author may draft the manuscript then circulate for feedback and revisions. When all authors have signed off on the final version of the manuscript, it is submitted to a journal.

For Paper Sprints to succeed, we have established a structured agenda for teams to follow that facilitates generating ideas, asking questions, and collaboratively making decisions. In this document, we have outlined principles and a detailed agenda to ensure a productive, collaborative Paper Sprint.

Principles of Paper Sprints

A Paper Sprint requires a safe, open environment to maximize the experience of working together. To develop this type of environment, the Paper Sprint is organized into a series of blocks during which the most salient elements of the manuscript workshopped by the group. By approaching each aspect of the project as a proposed idea, rather than a foregone conclusion, and doing this at the outset of the project, the team is able to implement feedback from the whole group for a truly collaborative research project.

To achieve this experience, Paper Sprints depend on several core principles:

Everyone Contributes

Information flow is often limited among team members when investigators write papers. The Paper Sprint provides a platform for everyone to contribute.

Everyone is Honest

The Paper Sprint provides an opportunity to not only look at the positive opportunities of a potential research project, but also why the project might fail. By looking at the strengths and weaknesses of the project, the team will be able to create a better project altogether. However, this can only be achieved if all participants express their perspectives honestly throughout the process.

Build on Each Other's Ideas

Academics often seek to find fault in ideas. At the same time, initial ideas often seem obvious or familiar. Instead of knocking down ideas, Paper Sprints seek to push the bounds of the team by building on these ideas. What is interesting or exciting about an idea? How might the idea be improved? Could you combine the ideas in a unique and novel way? This pattern of thinking will unlock new paths and opportunities for the team.

GETTING STARTED

Keep It Short

The Paper Sprint is intended to be a democratic forum where all participants have equal say in the direction of the research project. To achieve this, participants should be mindful about the length of their comments. Keep comments to two minutes to ensure everyone has an opportunity to provide their perspective.

By upholding these principles, the project team will achieve an engaging, productive dialogue that will lead to novel perspectives and mutual understanding about the manuscript and project plan.

Building the Team

A Paper Sprint ideally has a team of 4 to 7 people. The team should represent individuals with diverse perspectives on the research project.

Principal Investigator: The Principal Investigator contributes the paper topic and target for the Paper Sprint. The PI also plays the "Decider" role, which means that he or she makes the final decision about the direction of the paper. This role may be shared by the first author and senior authors.

Co-Investigators: The Co-Investigators provide added expertise to the research project.

Project Manager: The Project Manager will coordinate logistics (room, schedule, materials) and will ultimately keep everyone on track, ensure adherence to the timeline and assist with any barriers that arise. During the sprint, the Project Manager is responsible for note-taking and documenting all decisions.

Analysts: The Analyst will be able to provide added context about the data and analytical methods required for the project. After the Paper Sprint is done, he or she will also be performing the analysis specified during the sprint.

Students and/or Fellows: Students and/or Fellows often provide a novel perspective and important questions during the Paper Sprint. Sprints also present students and fellows with important learning opportunities, allowing them to observe how faculty approach answering research questions.

Facilitator: The facilitator ensures the team upholds the principles of the process, sticks to the timeline, and refocuses the conversation as needed. The Facilitator guides the team to reach their desired end goal. To do so, the Facilitator will lead the sessions, synthesize the conversation and maintain the democratic forum (i.e., make sure participants keep comments short by staying within their 2-minute limit).The Facilitator is most often the PI, but can also be the Project Manager, or another knowledgeable, objective participant.

Time, Space and Supplies

The Paper Sprint requires a two-hour session. Depending on the project, we may also schedule other meetings to (1) discuss details about the analysis, and (2) review results and write the manuscript (the Writing Sprint). While this may seem like a lot of time to commit, it's an opportunity to not only clarify your own thinking, but also synthesize your ideas with the team. We posit that you will achieve higher quality research projects in a more productive way by consolidating the time you spend into these sessions.

To get started, schedule the first two-hour time block with your Paper Sprint team. We recommend doing this at least two weeks in advance if not earlier to accommodate everyone's schedules.

In terms of space, you will need a room with a table for group discussions, as well as an open area with whiteboards, where it is easy for participants to move around. Ideally, the room should have a screen and/or projector that can be connected to a laptop, so that the team can view documents collaboratively. A second room, or quiet area, is helpful when the group splits up to write the abstract, but it not imperative.

We recommend a space that is different from where the team usually works together. The new environment can spark new ways of thinking. We have used the Collaboratory at the University of Michigan's Institute for Health Policy and Innovation as an effective collaborative space. A flat classroom with whiteboards and movable furniture can also be an effective space for the sessions.

In addition to whiteboards, the team will need whiteboard markers, blank sheets of 8.5″x11″ paper (printer paper), felt-tip medium-point markers, and tape (painter's tape and masking tape work best). Participants should all bring their laptops to collectively write and revise the abstract.

Note About Google Docs and Slack

The Paper Sprint is a collaborative project planning exercise. As such, we strongly recommend that the team use Google Docs to organize and store project materials, and ultimately write the paper. This will allow shared viewing, editing and commenting. In addition to Google Docs, we also recommend using Slack for team communications. This creates one central log of all project communication to which all team members have access. Google Docs and Slack can be synced for seamless document uploads to Slack with the ability to "refresh" documents after any updates have been made, reducing issues surrounding version control.

Resources

To guide the drafting of the manuscript, we have developed a manuscript-writing guide. The guide is a paragraph-level template that identifies the structure along with the content that is often included in these sections and suggested exhibits. It has been compiled from the rich set of manuscripts related to health policy and health services research that have been developed at the University of Michigan. In addition to this guide, participants also have access to a folder containing examples of relevant papers.

We also recommend that the team shares other material in advance of a sprint, including important literature or background materials. More on this in the next section.

Before the sprint

To prepare for the Paper Sprint, the first author in collaboration with co-investigators, develops a concept for the paper and, with the analyst, develops a proposed analysis plan. We have experimented with starting the Paper Sprint at different phases in the project arc and have found that the Paper Sprint is most productive when the idea is "baked enough" so that an analysis plan can be proposed.

Elements of the analysis plan include:

- Data sources
- Study population (inclusion and exclusion criteria)
- Study outcomes
- Exposure
- Statistical analysis
- Study design
- Level of analysis
- Modeling strategy
- Covariates
- Potential analytic challenges

A member of the team, whoever is best suited, compiles a list of literature relevant to the project into a Google Drive folder to share with the team. The team should review these papers in advance of the sprint.

Additionally, the first or senior author should identify who will write each section of the abstract. More on this later.

Lastly, if the group decides to hold a Writing Sprint, the first or senior will assign who will write and revise each section of the manuscript. More on this later, too.

On the next page is the overview of the Paper Sprint schedule. We will dive into the purpose and process of each session next.

Paper Sprint Schedule at a Glance

Setting the Stage — 10 min

Lead author provides background on the sprint
- + Title of paper
- + Target journal
- + Describe theory of paper, data and constraints
    - Knowledge gaps
    - Study data
    - Study cohort
    - Study design
- + What we will accomplish today

Group responds
- + Affirms or revises knowledge gaps
- + Asks big-picture questions about study approach

Writing the Abstract — 40 min

Use a structured abstract format (JAMA format)

Split into 2 teams
- + Intro team
- + Methods team

First round of writing (15 min)
- + Intro team writes Background and Objectives
- + Methods team writes Methods

First round of revision
- + Intro team revises Methods team’s work and vice versa

Second round of writing (15 minutes)
- + Intro team writes Conclusions
- + Methods team writes Results

Second round of revisions (5 minutes)
- + Intro team revises Methods team’s work and vice versa

Paper Sprint Schedule at a Glance

**Developing the Exhibits**	40 min	Using the manuscript guide as a model, all participants sketch out their plan for the exhibits (15 mins)<br><br>Exhibit drafts are taped to the walls and reviewed (5 mins)<br><br>Group discusses the best display for exhibits
**Determining the Analysis Plan**	20 min	Group reviews and comments on the analysis plan proposed (5 minutes)<br><br>Group discusses revisions to and questions about the analysis plan, one element at a time (15 minutes)
**Why Might We Fail?**	7 min	Group identifies enduring weaknesses and uncertainties<br><br>Develops a plan to resolve or acknowledge
**Determining Next Steps**	3 min	Lead Investigator will re-iterate next steps

Paper Sprint Schedule Step by Step

Setting the Stage

Team members introduce themselves and describe their role on the sprint team. The Facilitator (most likely the Lead Investigator) then provides an overview of the agenda and the principles of the sprint. The Facilitator will provide background on the project including the title of the paper, the theory behind the work, data to be used and any data constraints the project might be facing. If not already identified, the team will decide on a journal submission strategy at this time.

The Facilitator will then lead a discussion of the knowledge gaps, and how this project will fill those gaps. The group then has time to respond, affirming or revising those gaps. This is also a time for the group to ask big-picture questions about the study approach.

Writing the Abstract

The group will break into two teams (the Intro Team and the Methods Team) to write a structured abstract. We recommend the Journal of the American Medical Association (JAMA) style as it is easily adapted to other journal formats and lends itself well to the Paper Sprint process. Although the analysis has not been conducted, writing the abstract focuses the team and provides direction for the project. Placeholders are left for results to be inserted when the analysis is complete.

First, for 15 minutes, the Intro Team will write the Introduction section and the Methods Team will write the Methods section of the abstract. When the allotted time is up, the teams will switch and review each other’s work for 5 minutes. Then, for another 15 minutes, the Intro Team will write the Conclusions section and the Methods Team writes the Results section. Again, the teams swap and revise each other’s work for 5 minutes.

THE PAPER SPRINT

Developing the Exhibits

During this phase of the sprint, the team collaboratively develops the plan for the tables and figures. Each team member gets 5 sheets of blank 8.5″x11″ paper (i.e. standard printer paper) and a felt-tip medium-point pen. For 15 minutes everyone sketches their ideas for the paper's tables and figures, one table or figure per sheet of paper. Team members are encouraged to draw on the manuscript writing guide and the relevant literature circulated in advance of the sprint for inspiration.

After 15 minutes, the sketches are taped to the wall, reviewed, and discussed. The group decides which exhibits are best suited for the manuscript and should be included, as well as any modifications the group thinks would enhance the chosen tables and figures. We have found that taping the exhibit sketches to a dry erase board works well because it allows for the group to make notes around each exhibit, and even develop new sketches as a group.

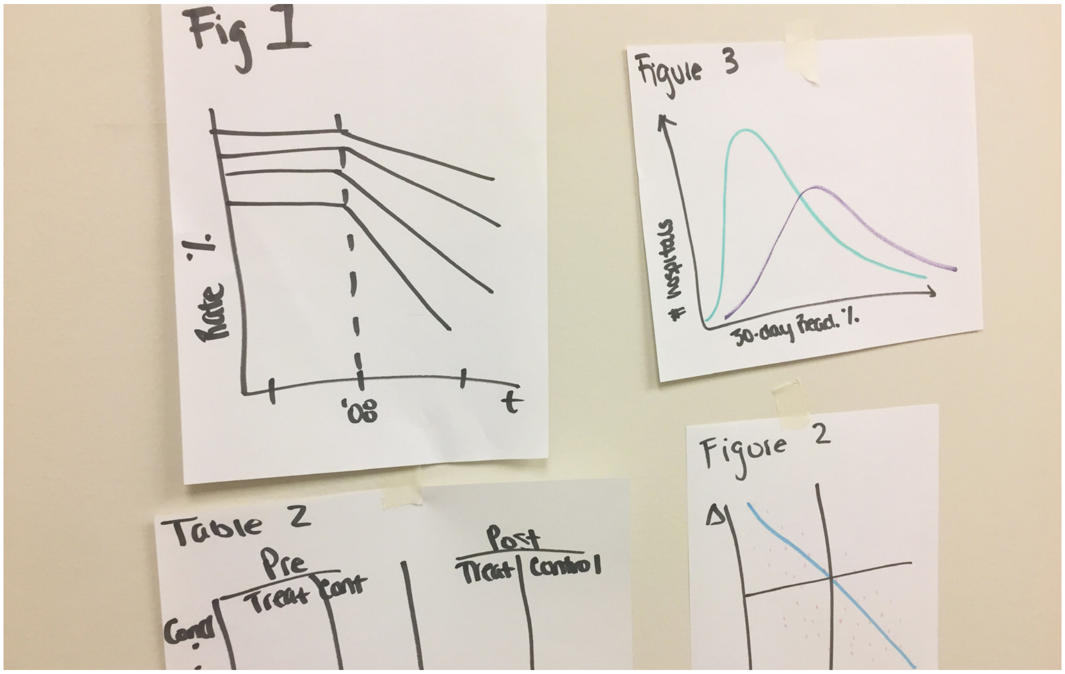

THE PAPER SPRINT

Determining the Analysis Plan

Using the exhibits to guide the conversation, for 5 minutes, the group will review the analysis plan proposed by the Analyst and the Lead Investigator. One element at a time, the group spends 15 minutes discussing revisions and resolving questions surrounding the analysis plan.

By the end of this session, the group will have specified a detailed analysis plan including methods, models, inclusion and exclusion criteria, outcomes, exposures, and control variables. If done well, the Analyst should have complete information at the end of this session to complete the analysis for the manuscript.

Why Might We Fail?

At this point, the group is asked to step back and think critically about the project they have developed and planned. The team should discuss and identify the top three reasons why the finished manuscript would be rejected from a journal. Once these top reasons are identified, the group works through each reason to either develop a solution, or acknowledge it as an inherent limitation.

Determining Next Steps

For the last 3 minutes, the Facilitator re-iterates what the team has accomplished and what the next steps are. Each team member should leave the sprint with a clear understanding of the project timeline and his or her expected contributions going forward. If the group plans to conduct a Writing Sprint, we recommend taking this time to schedule that session while everyone is in the room and can provide their availability. Typically, Writing Sprints occur 4 to 6 weeks after the Paper Sprint, which helps the group maintain focus and forward momentum on the project.

If the group will not be holding a Writing Sprint, the project plan should include who will be responsible for drafting the manuscript, circulating it for feedback, incorporating revisions and ultimately submitting the manuscript.

During the sprint, the Project Manager should be taking notes for the group and documenting the meeting, especially all decisions made. Additionally, the Project Manager retains copies of the selected exhibits, photographs them, and uploads the photos to the Google Drive project folder for the team’s reference. We also recommend photographing any notes written on the white board and uploading them to the Google Drive as project documentation.

(Optional) Writing Sprint Schedule at a Glance

**Setting the Stage**	5 min	Lead author describes the following:<br>– Justification for the paper<br>– Data<br>– Analysis<br>– Submission strategy
**Exhibit Review**	15 min	Review each exhibit in turn and discuss any revisions to the exhibits.
**Identifying the Narrative**	10 min	Review the narrative. Identify the key conclusions and supporting evidence for each conclusion.
**Discussing the Limitations**	5 min	Discuss limitations related to internal validity (i.e. why might our conclusions not be true?).<br>Discuss limitations related to external validity (i.e. why might our conclusions not generalize?).
**Discussing the Implications**	5 min	Identify the implications for each stakeholder and/or interest party.
**Draft Session**	45 min	Participants are each assigned a section of the manuscript and draft corresponding components of the manuscript.
**Revision Session**	25 min	Rotate participants to review Draft Session components.
**Conclusion and Next Steps**	10 min	Determine next steps for continued revision and submission, and assign who is responsible for these actions.

Writing Sprint Step by Step

When the analysis is complete the team can come back together to write! The Writing Sprint will build from the abstract created during the Paper Sprint.

SETTING THE STAGE

The PI starts the Writing Sprint by describing the justification for the paper, reminding the group of the relevant literature, and how this work fills a gap. Then, he or she describes the data and analysis performed for the project, but not the results. Finally, the submission strategy is reviewed and agreed upon. Typically, the group identifies a second and third journal for submission so that a plan is already in place if the manuscript is rejected.

EXHIBIT REVIEW

The team reviews all of the completed exhibits that have been created for the article, using the following questions as a guide for discussion:

- Are the results displayed clearly?
- Is there anything that is uncertain?
- Is there anything that should be added?
- Is there anything that should be removed?
- Are there any numbers that look strange?

The group identifies any revisions to the exhibits that would be necessary prior to submission.

IDENTIFYING THE NARRATIVE

Next, the group reviews the narrative. Does the team agree that the analysis leads to the conclusions made? The team should systematically work through each conclusion made and identify the evidence in the analysis that supports this conclusion. Finally, identify the "big takeaway" conclusion.

DISCUSSING THE LIMITATIONS

Now that the conclusions and exhibits have been identified and discussed, the group should step back and think about the limitations of the study. Examine limitations related to both internal and external validity:

- Internal Validity – Why might our conclusions not be true?
- External Validity – Why might our conclusions not generalize?

Discuss and identify the limitations of the work and how each should be addressed in the manuscript.

DISCUSSING THE IMPLICATIONS

Finally, before moving into writing, the team needs to think about the implications of this study. What does it mean? And for whom? Start by identifying the stakeholders, those affected, and interested parties or organizations. Then, in turn, discuss what the implications are for those groups.

DRAFT SESSION

With the narrative firmly established, your team is ready to write. During this session, the team will draft each section of the manuscript. The PI will work with the Project Manager to identify what the sections will be and assign 1-2 people to draft each section.

While there are no hard and fast rules to assigning participants to each section, you may want to choose individuals who are best informed about each section to write them. For example, the quantitative experts would be best equipped to write the methods and data sections. Depending on the number of participants in the session, the specific sections addressed may be modified.

WRITE

Next, each team member will spend 45 minutes drafting their assigned section. Participants should use the notes from the Paper Sprint and the completed analysis to guide their writing. While writing, participants should identify additional questions to be clarified with the team. These questions can be added as comments to the manuscript document.

REVISION SESSION

Team members will now rotate from the section that they wrote to a new section to revise. The team will spend 25 minutes revising their assigned section.

CONCLUSION AND NEXT STEPS

Now, the team has in hand a well-developed draft of their manuscript. To wrap up, the team should discuss any outstanding items that need to be addressed, as well as who is assigned to complete these tasks. For example, these might include:

- Are there any additional uncertainties about the analysis/interpretation that need to be resolved?
- Cover letter for submission
- Should the manuscript be circulated to any others who were not present at the Writing Sprint?
- Are there any obstacles to finalizing the manuscript?

Finally, the Principal Investigator should share the next steps for completing and submitting the manuscript.

Congratulations! You've finished the Paper Sprint! We hope that you've found this experience fun, productive and engaging with your team!
